# Langerhans Cell Histiocytosis in an Infant Mimicking a Lymphoma at Presentation

**DOI:** 10.1155/2015/670843

**Published:** 2015-10-26

**Authors:** Anjan Madasu, Asim Noor Rana, Saleh Banat, Hani Humad, Rashid Mustafa, Abdulrahman Mohd AlJassmi

**Affiliations:** ^1^Pediatric Hematology and Oncology Unit, Dubai Hospital, Dubai, UAE; ^2^Radiology Department, Dubai Hospital, Dubai, UAE

## Abstract

Langerhans cell histiocytosis (LCH) is a rare disorder characterized by proliferation and accumulation of clonal dendritic cells with varied clinical presentation and an unpredictable course. We report a 5-month-old infant with LCH who presented with severe respiratory distress, a large mediastinal mass, significant generalized lymphadenopathy, and hepatosplenomegaly. Lymphoma, especially T cell lymphoblastic lymphoma, can present with superior mediastinal syndrome needing urgent empirical therapy without biopsy. However, lack of response prompted a biopsy which confirmed it to be a case of LCH and that leads to appropriate therapy and survival. There have been reports of LCH presenting with isolated mediastinal mass or with generalized lymphadenopathy, but the combined presentation of generalized lymphadenopathy with large mediastinal mass, hepatosplenomegaly, and fever in an infant has rarely been reported. *Conclusion*. LCH should also be considered in the differential diagnosis of an infant presenting with generalized lymphadenopathy, mediastinal mass, hepatosplenomegaly, and fever.

## 1. Case History

We report a 5-month-old female infant who presented to us with progressive respiratory distress of 2-week duration, requiring ICU admission and supplemental oxygen. On examination, she was in severe respiratory distress with SpO_2_ of 82% requiring supplemental oxygen to maintain saturations above 94%. She had fever and generalized significant lymphadenopathy (3-4 cm in size) involving bilateral cervical, axillary, and inguinal areas.

**Figure 1:**
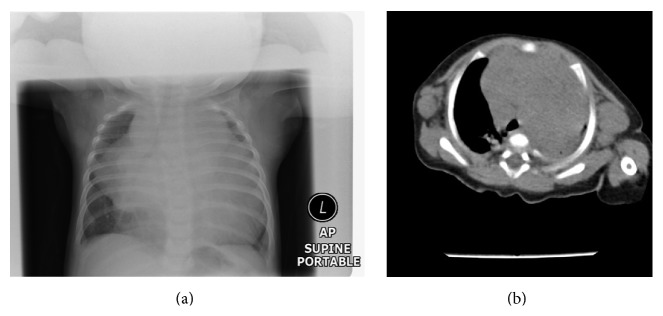
X-ray and CT scan of chest on presentation showing a large mediastinal mass with axillary lymphadeonpathy.

X-ray (Figure 1(a)) and CT scan of chest (Figure 1(b)) showed a large soft tissue shadow in the anterior mediastinum, anterior medial right thorax, and subtotal left thorax with compression of the left lung. Liver was palpable 10 cm below right costal margin and spleen was palpable 2 cm below the left costal margin.

The initial blood work showed WBC of 1.1 × 10^3^/*μ*L, Hb of 18.8 g/dL, and platelets of 326 × 10^3^/*μ*L. Blood film had left shift with toxic granulations and no blasts. With the probable diagnosis of leukemia/lymphoma, broad-spectrum antibiotics and IV Hydrocortisone were started.

**Figure 2:**
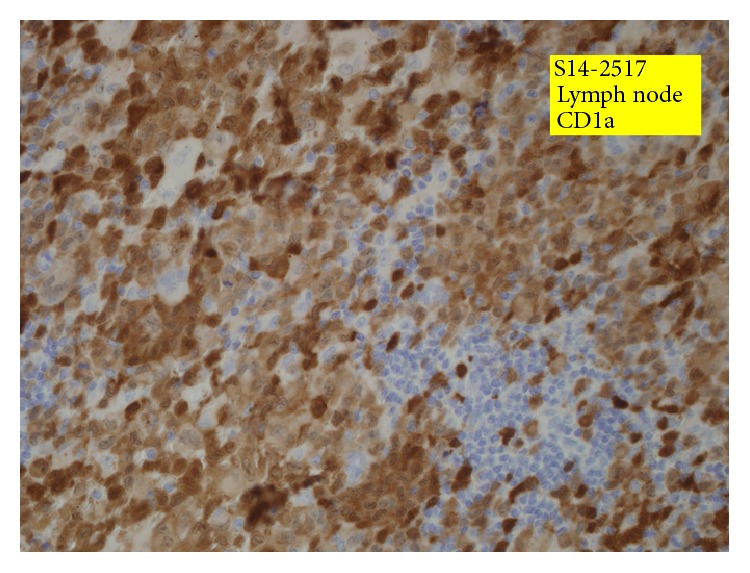
Lymph node showing CD1a positivity of tumor cells.

At 24 hours of admission, there was a maculopapular rash which progressively became more severe involving the major portion of her trunk, extremities, and palms. At 72 hours of admission, bone marrow examination done did not show any malignant infiltration. Biopsy of the left axillary lymph node and the skin done in the same setting was consistent with Langerhans cell histiocytosis as S100 and CD1a colabeled the tumor cells (Figure 2). Subsequently a skeletal survey as a work-up for LCH was done, but it did not show any osteolytic lesions. She fulfilled the criteria of multisystem LCH with skin, lymph node, liver, and spleen involvement. She was commenced on chemotherapy as per LCH-IV protocol with weekly Vinblastine (3 mg/m^2^) and Prednisolone (20 mg/m^2^), which was 50 percent of the standard dose as her age was below 6 months.

At 120 hours of admission, the infant developed severe respiratory distress in spite of chemotherapy and was hence ventilated. As she was requiring high ventilator settings with no improvement in the chest radiography, she was pulsed with Methylprednisolone at 20 mg/kg for 3 days and Etoposide at 75 mg/m^2^ was later added when there was still no clinical or radiological improvement. A second dose of Etoposide was also given after 7 days of the first dose as there was no improvement in clinical or ventilator parameters.

At day 15 of chemotherapy, the infant started responding and could finally be extubated.

**Figure 3:**
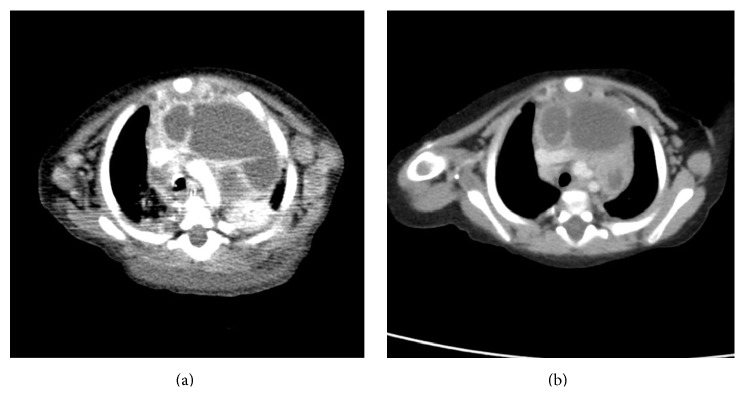
CT scan of chest showing reduction of mediastinal lesions.

CT scan (Figure 3(a)), done in the 3rd week of therapy, showed some response with chemotherapy in the form of necrosis in the lymph nodes with marginal decrease in size. An evaluation after 6 weeks of induction chemotherapy showed that she fulfilled the criteria of “active disease better” and hence reinduction chemotherapy was given for another 6 weeks.

After the 12 weeks of initial chemotherapy, CT scan (Figure 3(b)) showed further reduction in the size of the previously seen anterior mediastinal soft tissue mass and remarkable reduction in the size of the multiple abdominal, pelvic, and inguinal lymph nodes and disappearance of the pericardial and pleural effusion. Hence, she now fulfilled the criteria of “active disease with regression of lesions.”

**Figure 4:**
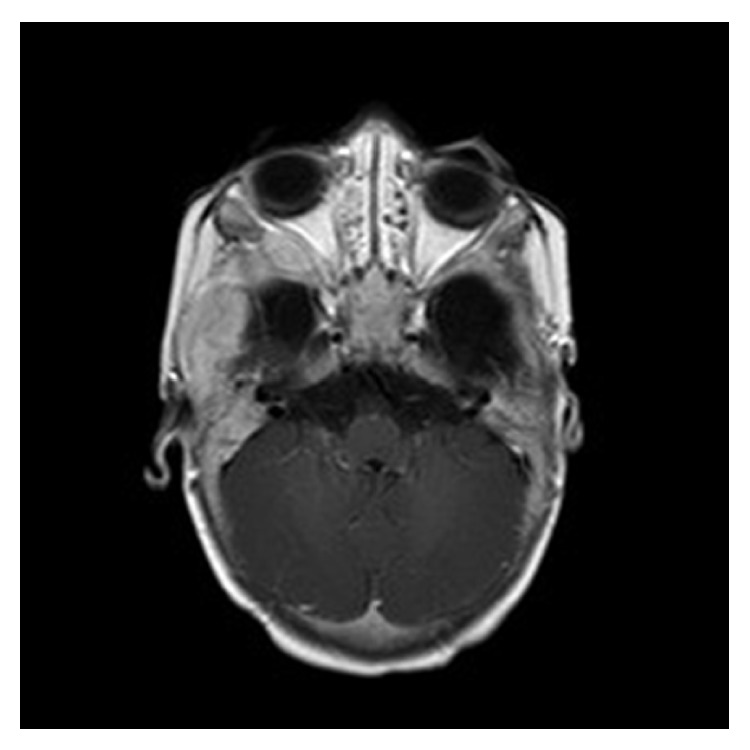
MRI of face showing soft tissue mass in the left mastoid region with osteolytic lesion in the greater wing of sphenoid bone, left temporal, and right temporal regions.

While she was about to start the continuation therapy, she was again readmitted with complaints of fever and swelling of right temporal area with otorrhoea. An MRI of brain (Figure 4) done showed soft tissue mass in the right temporal region with osteolytic lesion in the greater wing of the right sphenoid bone and involvement of both temporal bones which was further confirmed by PET/CT scan done on 28 August 2014. A few necrotic lesions were still present in the mediastinum, which had significantly reduced in size but were not PET avid.

**Figure 5:**
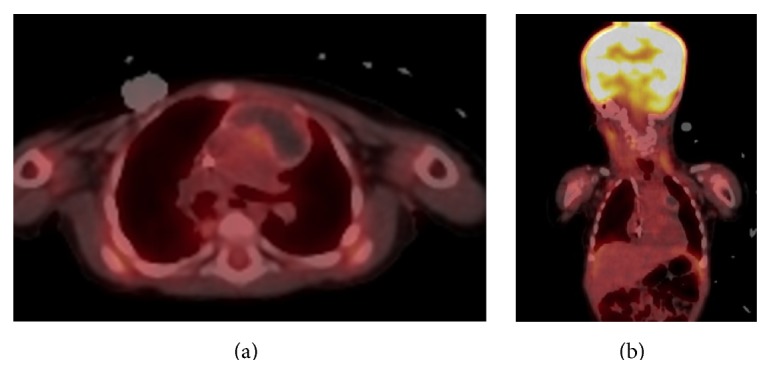
Whole body PET/CT scan showing no significant metabolically active lesions.

**Figure 6:**
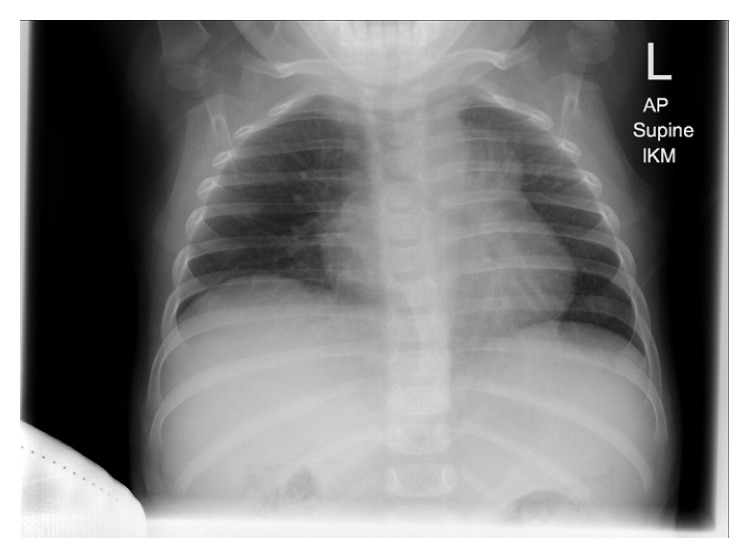
X-ray of chest showing resolution of the previously seen mediastinal mass, except for a small left parahilar opacity.

Since all other previous lesions regressed dramatically with the new temporal region lesions, she was classified into active disease at the end of stratum 1. She was hence started on stratum 2 chemotherapy as per LCH-IV protocol, which consisted of Prednisolone, Cytosine Arabinoside, and Vincristine for a 24-week period. Since Cladarabine or Clofarabine was not readily available with us, we preferred to go ahead with stratum 2 therapy to see the response. At the end of stratum 2 therapy, PET/CT scan was repeated on 19 February 2015. Though the PET/CT scan of lung window (Figure 5(a)) demonstrated some residual necrotic lesions, these were not metabolically active. As there was good PET response (Figure 5(b)), with no progressive lesions or new active lesions, she was put on continuation therapy. A chest X-ray (Figure 6) done at 3 months of continuation therapy shows a significant resolution of the previously seen mediastinal mass, except for a small left parahilar opacity. This opacity could still be the remnant of nonactive mediastinal shadow, which was seen in the PET scan dated 19 February 2015.

BRAF and MAP2K1 mutations could not be done, as we do not have such facilities available with us.

## 2. Discussion

LCH has a wide spectrum of disease activity, ranging from single osteolytic lesion to rapidly fatal leukemia-like illness. Commonly involved systems are skin, bone, lymph nodes, central nervous system (including diabetes insipidus), ears, gums, and lungs. The extent of disease is staged as follows: (1) single-system disease; (2) multisystem disease; (3) multisystem disease with risk organ involvement [1].

Though many cases of LCH have been reported to have mediastinal mass [2–6], the combination of generalized lymphadenopathy with large mediastinal mass in multisystem LCH is an unusual presentation in an infant and has rarely been reported.

Our child clearly illustrates that generalized lymphadenopathy with large mediastinal mass can be a presentation of LCH in infants causing significant respiratory compromise because of major airway compromise in infants. Such presentation can be seen in hematological malignancies, germ cell tumors, or neuroblastoma in children.

LCH must be excluded in the differential diagnosis of such presentations, as an early diagnosis is required to plan for further therapy.

Standard induction therapy of Prednisone and Vinblastine may not be sufficient to control the disease, especially in aggressive forms of LCH, which has been demonstrated in our child. Addition of Pulse Methylprednisolone and Etoposide was successful in reducing the mediastinal mass, which helped us in extubating the child. Gadner et al. have already reported the benefits in increasing treatment intensity in LCH with risk organ (RO^+^) involvement. In a randomized controlled trial involving 193 children, the group showed that addition of Etoposide 150 mg/m^2^ every week for six weeks to Vinblastine and Prednisolone increased rapid responses in RO^+^ group from 43% to 68% and reduced mortality from 44% to 27% [7].

Our child had persistence of residual necrotic mediastinal lesions at the end of 9 months of therapy. The last PET/CT scan dated 19 February 2015 demonstrated that there was good response to the chemotherapy with no evidence of progressive disease, which helped us proceed with continuation therapy. The continuation therapy would be oral 6-mercaptopurine, 50 mg/m^2^ daily, and oral methotrexate, 20 mg/m^2^ weekly, for a period of 24 months. PET scans have been proven to be the most sensitive functional test in identifying LCH lesions and evaluating therapy response [8, 9]. PET/CT scan certainly helped us plan further course of therapy.

## 3. Conclusion

We conclude that LCH should also be considered in the differential diagnosis of an infant presenting with generalized lymphadenopathy, mediastinal mass hepatosplenomegaly, and fever. An early diagnosis and proper treatment can achieve good outcome in such children. Our child is well now and is asymptomatic and completed 7 months of continuation therapy.
